# Delayed Psychological Morbidity Associated with Snakebite Envenoming

**DOI:** 10.1371/journal.pntd.0001255

**Published:** 2011-08-02

**Authors:** Shehan S. Williams, Chamara A. Wijesinghe, Shaluka F. Jayamanne, Nicholas A. Buckley, Andrew H. Dawson, David G. Lalloo, H. Janaka de Silva

**Affiliations:** 1 Department of Psychiatry, Faculty of Medicine, University of Kelaniya, Ragama, Sri Lanka; 2 Department of Medicine, Faculty of Medicine, University of Kelaniya, Ragama, Sri Lanka; 3 South Asian Clinical Toxicology Research Collaboration, University of Peradeniya, Peradeniya, Sri Lanka; 4 Prince of Wales Clinical School, University of New South Wales, Sydney, Australia; 5 Liverpool School of Tropical Medicine, Liverpool, United Kingdom; Universidad de Costa Rica, Costa Rica

## Abstract

**Introduction:**

The psychological impact of snakebite on its victims, especially possible late effects, has not been systematically studied.

**Objectives:**

To assess delayed somatic symptoms, depressive disorder, post-traumatic stress disorder (PTSD), and impairment in functioning, among snakebite victims.

**Methods:**

The study had qualitative and quantitative arms. In the quantitative arm, 88 persons who had systemic envenoming following snakebite from the North Central Province of Sri Lanka were randomly identified from an established research database and interviewed 12 to 48 months (mean 30) after the incident. Persons with no history of snakebite, matched for age, sex, geograpical location and occupation, acted as controls. A modified version of the Beck Depression Inventory, Post-Traumatic Stress Symptom Scale, Hopkins Somatic Symptoms Checklist, Sheehan Disability Inventory and a structured questionnaire were administered. In the qualitative arm, focus group discussions among snakebite victims explored common somatic symptoms attributed to envenoming.

**Results:**

Previous snakebite victims (cases) had more symptoms than controls as measured by the modified Beck Depression Scale (mean 19.1 Vs 14.4; p<0.001) and Hopkins Symptoms Checklist (38.9 vs. 28.2; p<0.001). 48 (54%) cases met criteria for depressive disorder compared to 13 (15%) controls. 19 (21.6%) cases also met criteria for PTSD. 24 (27%) claimed that the snakebite caused a negative change in their employment; nine (10.2%) had stopped working and 15 (17%) claimed residual physical disability. The themes identified in the qualitative arm included blindness, tooth decay, body aches, headaches, tiredness and weakness.

**Conclusions:**

Snakebite causes significant ongoing psychological morbidity, a complication not previously documented. The economic and social impacts of this problem need further investigation.

## Author Summary

Snakebite envenoming is a neglected public health problem, especially in rural areas of tropical and sub-tropical countries. Little is known about the long term effects, and even less about the possible psychological effects, of snakebites and envenoming. We investigated the possible psychological impact of snakebite in 88 persons who had been envenomed 1 to 4 years ago in a rural agricultural area in Sri Lanka by using accepted measurements of psychological disability and group discussions among victims. 88 persons from the same areas who had not been bitten by a snake, but were of similar age, sex and occupation were also assessed in a similar manner. Compared to those who had not been bitten, snakebite victims had significantly more symptoms suggesting psychological disability, depression and post-traumatic stress. More than a fourth of those bitten claimed that the snakebite caused a negative change in their employment, and 10% had stopped working altogether. 17% claimed to have residual physical disability which they attributed to the bite, although no disability could be detected when they were examined. These findings indicate that snakebite results in ongoing psychological disability even 1 to 4 years after the episode, a complication that has not been previously reported.

## Introduction

Snakebite is a significant health issue in the rural tropics. Globally, it has been estimated that at least 421,000 envenomings and 20,000 deaths occur due to snakebite each year each year, and that these numbers may even be as high as 1.8 million envenoming and 94 000 deaths [1]. The highest burden exists in South Asia, Southeast Asia, and sub-Saharan Africa. In Sri Lanka, about 40000 persons were treated for snake bite in government hospitals each year [2]. The actual number of bites is likely to exceed this number, as many of the victims seek traditional forms of treatment. Snakes are feared for their bite associated mortality and morbidity but only six of the 92 snake species in Sri Lanka are medically important. These are the Russell's viper, cobra, the two kraits (common and Sri Lankan), saw scaled viper and hump nosed viper. The Russell's viper, cobra and kraits account for most of the morbidity and mortality.

Most studies on snakebite only estimate the numbers of snakebites, acute complications and deaths[3]. There is very little data on the long term physical and psychological consequences experienced by victims of snakebite. This is unfortunate, as most snakebite victims are in the economically productive age group, and the economic impact of any disability is likely to be high. Snakebites are sudden and unexpected, and the element of surprise and the associated threat to life may cause extreme stress and anxiety in the victim. The long term psychological consequences of this, such as, post-traumatic stress disorder, generalized anxiety, avoidance of situations where they could be bitten again, health seeking behaviour and somatisation have not been previously studied. The objective of our study was to assess stress and anxiety: particularly symptoms of anxiety and depression, post-traumatic stress disorder, somatisation and impairment in functioning, at least 12 months following snakebite envenoming.

## Methods

### Setting and context

The Polonnnaruwa district of the North Central Province in Sri Lanka was selected for the study. This is in the dry zone with a predominantly rural agricultural population. The highest numbers of snakebite envenoming in Sri Lanka are reported from this region [3]. Mental health services in this area are poorly developed. The research team had access to an established database of snake bite victims from a previous study conducted in this area.

### Design

There was a quantitative arm and qualitative arm to the study.

In the quantitative arm 200 persons (cases) over 18 years of age, with a history of snakebite envenoming which had required treatment with antivenom at least 12 months previously, were randomly selected by the computer from among 296 eligible persons included in a database of 1500 snakebite victims from a previous study conducted in the area. Initial sample size calculation, based on 10% baseline anxiety and depression rate in the community and an expected doubling in the rate of depression and anxiety to 20% in snake bite victims (at 80% power and 5% significance level), showed the need for 90 participants. Allowing for a more than 50% attrition rate, considering the time period since the bite and poor transport infrastructure in these rural areas, we planned to randomize 200 snakebite victims. Letters (in Sinhala – the North Central Province is mainly inhabited by a Sinhala literate population) were sent out to those selected inviting them to participate in the study.

They were assessed by medical officers using a structured questionnaire on demographic characteristics, circumstances of the snake bite envenoming, hospital stay, perceived severity of the bite, and return to work and functioning. A physical examination was conducted to assess disability related to the snake bite.

The following measures were administered to quantify psychological distress: a modified Sinhala version of the Beck depression inventory [4], Post-traumatic Stress Symptom Scale – Self Report (PSS-SR) [5], the Hopkins symptoms checklist – 25(HSCL-25) [6], [7] and the Sheehan Disability Inventory [8] which have all been previously validated and used in Sri Lanka [9]. The psychological scales were administered by psychiatrists with knowledge and experience in administering these tools.

Local hospital attendees matched for age, sex, geograpical location and occupation and without a history of snakebite were invited as controls. They were also administered the modified Sinhala version of the Beck depression inventory, a modified Hopkins somatic symptoms checklist and Sheehan Disability Inventory.

In addition, focus group discussions were held with snakebite victims to explore perceived long term effects of the snakebite. A total of five focus group discussions were held until data saturation occurred. Each group consisted of 6–10 snake bite victims and was initiated by the same moderator who raised the question of possible long term health effects of the snake bite. The moderator did not participate in the discussion apart from clarifying unclear statements and facilitating the discussion. The group members discussed what they perceived to be effects on their health. The discussions were tape recorded and transcribed by independent evaluators.

### Ethical considerations

Informed written consent was obtained from all participants. Those identified to have severe psychological distress were referred to appropriate psychiatric services for further assessment and follow up. Ethical approval was obtained from the Ethics Committee of the Faculty of Medicine, University of Kelaniya, Sri Lanka.

### Data Analyses

Analysis of quantitative data was done using SPSS version 16. Comparisons between cases and controls were made using non-parametric tests. The Beck's modified depression scale scores were categorized into no depression (0–15), mild depression (16–24), moderate depression (25–32) and severe depression (>32) in terms of accepted figures. The established clinically significant item-average cut-off score of ≥1.75 for each sub-scale was used for the Hopkins somatic symptoms checklist. The accepted cut off score ≥20 on the PSS-SR was taken as compatible with post traumatic stress disorder. Spearman correlations were used to compare the different symptoms scales and the factors within them. Chi squared test was used where appropriate for categorical variables. Qualitative data were thematically analyzed.

## Results

### Response rate

Of the 200 snake bite victims (167 males, 33 females) to whom the letters of invitation were sent, 88 (74 males, 14 females) responded and participated in the study. The mean age of the responders was 41.6 (SD 13.7) years compared to 37.5 (SD 12.7) (P<0.013) in the non-responders. There was no statistically significant difference in, sex, occupational status, ethnicity, mean duration of hospital stay, treatment with antivenom or severity of reaction to antivenom between responders and non-responders (Table 1). The majority in both groups were unable to identify the offending snake.

**Table 1:** Characteristics of the snakebite victims who responded to the invitation and non-responders.

	Responders	Non responders	Significance
**Sex – Males (%)**	74 (84)	93 (83)	NS
**Mean age (SD)**	42.6 (13.7)	37.5 (12.7)	P = 0.013
18–30 yrs (%)	23 (26)	39 (35)	
31–45 yrs (%)	26 (29.5)	42 (37.5)	
46–60 yrs (%)	30 (34)	25 (22.5)	
61 yrs and above (%)	9 (10.5)	6 (5)	
**Occupation**			
Farmers (%)	42 (47)	56 (50)	NS
Unskilled workers (%)	22 (25)	22 (20)	NS
Skilled workers (%)	17 (19)	21 (19)	NS
Teachers (%)	1 (1)	3 (2.5)	NS
Office workers (%)	2 (2)	3 (2.5)	NS
Military personnel (%)	4 (4.5)	7 (6)	NS
**Ethnicity – Sinhala Buddhist (%)**	81 (92)	105 (94)	NS
**Mean duration of hospital stay in days (SD)**	4.2 (SD 1.8)	4.5 (SD 2.2)	NS
**Snake identified (%)**	20 (23)	29 (26)	NS
**Snake envenoming/antivenom administration (%)**	88 (100)	112 (100)	NS
**Severity of reaction to antivenom**			
No reaction (%)	30 (34)	42 (37.5)	NS
Mild (%)	4 (5)	6 (5)	NS
Moderate (%)	22 (25)	31 (27)	NS
Severe (%)	32 (36)	33 (29.5)	NS

NS – not significant.

### Depression score

The mean depression score in the cases[19.1 (SD 7.7)] was significantly higher than that of controls [14.4(SD 2.5)] [p<0.001; mean difference 4.74 (95%CI 3.02–6.46)] (Table 2). In terms of these scores, 48 (54%) cases and 13 (15%) controls met criteria for depressive disorder. Similarly the Hopkins symptoms checklist score [38.9(SD 16.3)] in cases was significantly higher than that of controls **[**28.1 (SD 5.8)] [p<0.001; mean difference 10.735 (95% CI 7.06–14.41)]. The depression subscale scores in the Hopkins checklist showed that 20 (23%) of cases and two (2.3%) of controls were depressed. The correlation between the modified Beck depression score and the Hopkins anxiety score (r = 0.728; p<0.001) and Hopkins depression score (r = 0.856; p<0.001) were highly significant. On multiple regression analysis none of the variables, namely, age, sex, occupation, duration of hospitalization, ICU admission and adverse reactions to antivenom predicted depression.

**Table 2:** Characteristics of snakebite victims (cases) and controls.

	Cases	Controls	Significance
**Sex**	M 74 : F 14	M 74 : F 14	NS
**Mean Age (SD)**	41.6 (13.7)	42.2 (12.1)	NS
**Occupation – Farmers (%)**	42 (47)	37 (42)	NS
**Modified Beck Depression - mean (SD)**	19.1 (7.7)	14.4 (2.5)	P<0.001
Number depressed (%)	48 (54)	13 (15)	P<0.0001
Mild (%)	34 (38.6)	12 (13.6)	
Moderate (%)	7 (8.0)	1 (1.1)	
Severe (%)	7 (8.0)	-	
**Hopkins anxiety score – mean (SD)**	16.6 (6.9)	11.4 (2.3)	P<0.001
**Hopkins depressive score – mean (SD)**	22.2 (10.0)	16.7 (3.8)	P<0.001
Depressed (%)	20 (23)	2 (2.3)	P<0.001
**Hopkins score total - mean (SD)**	38.9 (16.3)	28.1 (5.8)	P<0.001
Psychiatric morbidity (%)	31 (35)	4 (4.7)	P<0.001
**PTSD score - mean (SD)**	10.5 (12.7)	-	
Number with PTSD (%)	19 (21.6)	**-**	
**Sheehan disability score(SD)**	13.66 (10.89)	2.92 (3.63)	P<0.001
**Residual physical disability (%)**	15 (17)	**-**	
**Negative effect on employment (%)**	24 (27)	10 (12)*	P = 0.007
**Stopped working (%)**	9 (10.2)	3 (3.5)*	P = 0.07

*Negative effect on employment or stopped working due to any reason over the preceding three years.

NS – not significant.

### Post-traumatic stress disorder (PTSD) score

The mean post-traumatic symptom scale score among cases was 10.5 (SD 12.7). Nineteen cases (21.6%) met criteria PTSD. The total PTSD score correlated strongly with the disability scores and the depression and anxiety scores (Table 3). PTSD was a significant predictor of depression on the modified Beck depression score [P = 0.004; Odds ratio 9.828 (95% CI 2.1–41.8)].

**Table 3:** Correlation with post-traumatic stress disorder symptom (PTSD) score.

	PTSD Score (n = 88)	
	Pearson Correlation	(Sig. (2-tailed)
**Disability score**	0.648**	0.000
**Hopkins anxiety score**	0.759**	0.000
**Hopkins depression score**	0.823**	0.000
**Hopkins total score**	0.829**	0.000
**Beck score**	0.728**	0.000

**Correlation is significant at the 0.01 level.

The symptoms contributing most to the PTSD score were avoidance behavior (r2 = 0.845), hypervigilance (r2 = 0.826) and physical changes related to hyperarousal (r2 = 0.843). However all the symptoms showed good correlation with the total PTSD score. The mean values of the PTSD measure were significantly higher in females (19.93) compared to males (8.72) [p<0.005, mean difference 11.21]. On multiple regression analysis age, sex, occupation, duration of hospitalization, ICU admission and adverse reactions to antivenom did not predict PTSD.

### Disability and effect on employment

The Sheehan disability inventory showed a significant difference between cases and controls (13.66 vs 2.99; p<0.001; mean difference 10.74; 95% CI 1.25–13.25). 17% of the cases claimed to have residual physical disability despite there being no external evidence on physical examination.

A negative effect on their subsequent employment resulting in less skilled or fewer hours of work was claimed by 24 (27%) of victims; nine (10%) had stopped working after the incident. In comparison, during the preceding three years, ten (11.9%) of the control group (P = 0.007) had a change of job resulting in less skilled employment or fewer hours of work and three (3.5%) (P = 0.07) of the control group had stopped work due to various reasons.

### Qualitative findings

Various physical symptoms were attributed to the snake envenoming. Five main themes were identified - poor vision, tooth decay, body aches, headaches, weakness and tiredness of the body. Poor vision, body aches and tiredness were the most frequently occurring observations. “My vision has become poorer. It is as if there is a net in front of my eyes …….”; “My eye sight fluctuates since this event. One day I can see clearly but on some days my vision is poor. As I'm a teacher I find these problems affecting my work and it's very difficult to teach anymore ….”; “I am having a thousand problems after the snake bit me. I have arm pain, stomach aches, eye pain, weakness of my legs, poor vision. I work in the fields but do so with great difficulty ….”.

Some interesting rare comments were bordering on overvalued or delusional ideas - “After getting bitten by the snake I feel a foul smell emanating from my sweat. I think this is the snake's venom leaving my body….”.

## Discussion

Our findings show significant psychological morbidity one to four years after snakebite envenoming – a hitherto unrecognised phenomenon. This study demonstrates depressive symptoms in more than 50% of snake bite victims who had been treated for serious envenoming, more than 1 year after the index episode. It is higher than the 15% seen in the control group, and the 10% baseline community prevalence predicted by the World Health Organisation [10]. Most reviews that emphasize the importance of snakebite related morbidity and mortality and its public health impact do not adequately address the issue of psychological morbidity [1], [11], [12]. More recently, however, the impact of poverty [13] and the need for research into long-term psychological effects of snakebite [14] have been highlighted.

The literature on psychological morbidity following animal bites is scarce. In a study from China, 19 out of 358 children developed PTSD following animal attacks [15]. In another study, 12 out of 22 children had symptoms of post-traumatic stress disorder two to nine months after a dog bite [16]. Our study is perhaps the first on the psychological consequences of animal bites in adults.

### Social disadvantage and stress

The high prevalence of psychological distress in the study population, including the control group, could at least partly be attributed to social disadvantages experienced by rural communities in developing countries. These were adults (mean age of 41 years), with young families, living in poverty with a daily income of less than US$7.50 and often working under difficult conditions in farms and rice fields. The snake bite may be the adverse life event that tips the balance [17], leading to psychological problems that persist long after the physical recovery. Associations between poverty and depression [10] and suicidal ideation [18], and even risk of PTSD [19], [20] have been previously documented. The additive effects of poverty and intimate partner violence in women with PTSD, depression and emotional difficulties have been discussed before [21]. Higher social support seems to predict lower PTSD severity at least for women with cumulative interpersonal trauma [22].

### Comparison with psychological morbidity after other trauma

Following the tsunami that affected Sri Lanka in 2004, PTSD and depression rates were 21% and 16% respectively, 20–21 months after the event [9]. In a study that looked at car crashes, as many as 23% of hospitalised passengers and 11% of hospitalised drivers were shown to have significant levels of stress 18 months after the incident [23]. Following war trauma in a civilian population in Sri Lanka, 27% reported PTSD, 25% major depression, 41% somatization and 26% anxiety disorders [24], [25]. The unadjusted weighted prevalence rate reported among mass conflict victims for PTSD was 30.6% (95% CI, 26.3%–35.2%) and for depression was 30.8% (95% CI, 26.3%–35.6%) [26]. The PTSD prevalence in our snakebite victims is comparable to the rates seen after the tsunami and car crashes, but lower than that reported following war trauma in Sri Lanka. In contrast, depression symptom scores were higher in snakebite victims. This might be explained by sub-threshold depressive symptoms or somatization, not meeting criteria for serious depressive disorder. However, in our study, at least 16% of victims met criteria for moderate to severe depressive disorder, as opposed to 1% in the control group. The more conservative estimate in the Hopkins depression sub-scale of depressive disorder in 23% of snakebite victims may reflect the true prevalence although it appears to underestimate the morbidity, as the controls too have a lower than expected percentage with depressive symptoms.

### Cultural perceptions

In psychological terms, a stressful event can be classified as a natural disaster. However, the event for the subject is individual and not collective akin to other natural disasters. The fear of death is very real and can lead to subsequent avoidance and phobic symptoms. Many people are terrified of snakes and the irrational fear called ophidiophobia persists despite most species being non-venomous. Beliefs and myths regarding snakes abound in many societies due to their characteristics such as speed and agility, the bifid tongue, unblinking lidless eyes, ability to renew their skin and inject venom. They have been objects of worship and awe as people attribute wisdom, cunning, power, fertility, sexuality and renewal of life to them, particularly in Africa and the Indian subcontinent [27]. In Sri Lanka too snakes are revered, and particularly the cobra is considered sacred. Stories of protection as well as vengeful attacks by snakes for past atrocities even in a previous birth, based on a belief of re-birth as animals, abound. These beliefs may colour the perceived long term effects of poor vision, weakness and fatigability brought out in the qualitative themes. In cognitive behavioural terms, the snakebite could be a critical incident, acting on existing psychological schemas, triggering negative automatic thoughts leading to anxiety and depression.

### Burden of disease

In Sri Lanka, the incidence of snakebite is highest in the rural, agricultural areas. As most bites occur outdoors, any avoidance behaviour associated with underlying psychological morbidity [16] could result in avoidance of work in the fields or on farms resulting in loss of income. This is compounded by the fact that few psychiatric services are available in these rural areas, and primary care physicians may easily miss any psychological morbidity associated with snakebite.

The attrition rate of more than 50% from the sample randomised is a significant limitation of this study. Those who participated in the study were older than the non responders, and we may therefore have ended up with a sample of older victims who were more maladjusted and had assumed a sick role after the envenoming. But even assuming a best case scenario with no morbidity among the non-responders the prevalence of depression would still be around 25% of the total population randomized, demonstrating a major burden of psychological ill health following snake bite. Probably due to our small sample we could also not find any predictors for depression and PTSD. Further exploration of the overall impact of snake bite in the rural tropics and the direct and indirect costs associated with the psychological sequelae and loss of employment is warranted [14], [28].
